# Restriction vs Constriction

**DOI:** 10.1016/j.jscai.2023.100965

**Published:** 2023-06-21

**Authors:** Larry S. Dean, Morton J. Kern

**Affiliations:** a University of Washington School of Medicine, Seattle, Washington; b VA Long Beach, Long Beach, California

**Keywords:** Constrictive pericardial disease, restrictive cardiomyopathy, hemodynamics

Clinical presentations of dyspnea, pedal edema, enlarged liver, and jugular venous distention may require differentiating constrictive pericardial disease (CPD) from restrictive cardiomyopathy (RCM). Differentiation of the pathophysiology is of paramount importance to the invasive cardiologist because they are often called to sort out a confusing clinical picture. The surgical treatment of pericardial constriction not only can be life changing for the patient but also involves a considerable risk.

Therefore, an accurate hemodynamic and echocardiographic evaluation is required, which will lead a patient’s care along the proper road; an erroneous diagnosis could lead to a dead end, futile treatment, or harm to the patient from omission of correct therapy. Using invasive hemodynamics to differentiate various forms of right atrial hypertension has been ongoing for decades.^1^, ^2^, ^3^, ^4^ As previously discussed,^5^ the initial steps in avoiding the wrong diagnosis include properly setting up the hemodynamic measurement system. Key points for success are as follows:

1. Correctly flushed tubing and zeroed transducers
2. Appropriate catheter selection
3. Simultaneous pressure measurements
4. Interpretation of the results

Now, we will cover each in more detail.

## Correctly flushed tubing and zeroed transducers

The set up for accurate hemodynamics was addressed in detail,^5^ but the fundaments for accurate measurements cannot be overlooked. Transducers should be zeroed at the same level and midchest. Otherwise, subtle differences in right ventricular (RV) and left ventricular (LV) pressures, such as equivalent end diastolic pressures, may be lost or misinterpreted.

## Appropriate catheter selection

Because the diagnosis of constrictive/restrictive physiology depends on simultaneous LV and RV pressures, when possible, catheters should be of equal length, similar compliance, and fidelity. A standard balloon-tipped pulmonary artery catheter for RV hemodynamics may not provide the fidelity required because the standard pulmonary artery catheter characteristics of high compliance and a small lumen often generate artifacts such as amplification of waveforms due to the single-end hole design.

Q: What is an ideal fluid-filled catheter?

• One with multiple holes. Example: a pigtail or multipurpose multiple side hole catheter
• One with a large lumen. Example a pigtail or >6F Wedge catheter (not a Swan-Ganz or multilumen catheter)

A: Properly placed pigtail catheters in both the right and left ventricles.

## Simultaneous pressure measurements

Both CPD and RCM impair diastolic filling through different mechanisms. Because CPD encases the heart, ventricular filling is fixed, and one ventricle fills at the expense of the other.

The relationship of RV and LV pressures during respiration is termed ventricular interdependence, and measurements demonstrating this phenomenon provide the highest sensitivity and specificity compared with historical hemodynamic findings purported to be useful in differentiating CPD from RCM. One famous hemodynamic wave form is the exaggerated rapid filling wave, followed by a flat period of diastasis, seen as a “dip and plateau,” commonly known as the square root sign. Unfortunately, the square root sign is not sensitive nor specific for CPD or RCM and has been noted in congestive heart failure, bradycardia, and RV infarction.

## Interpretation of the results

After placing a pigtail catheter in the right ventricle and a second catheter in the left ventricle, simultaneous RV/LV pressures can be measured in a sinus rhythm or a paced rhythm if the patient has underlying atrial fibrillation.^2^

**Figure 1:**
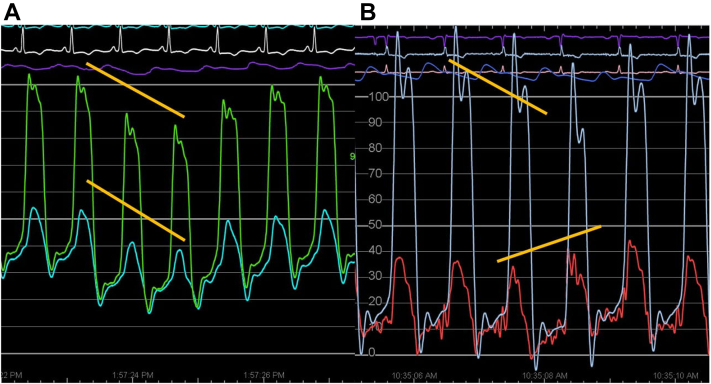
(**A**) Restrictive pattern showing concordance with inspiration. (**B**) Constrictive pattern showing discordance with inspiration. Note that both have a “dip-and-plateau” diastolic filling pattern.

Then, the continuous hemodynamics with the patient spontaneously breathing can be reviewed. During the inspiratory phase of respiration, if RV and LV waveforms rising and falling together (ventricular concordance) are noted and the RV and LV end diastolic pressures are elevated, then the RV/LV systolic pressure concordance is most consistent with restrictive physiology (Figure 1A).

On the contrary, if during the inspiratory phase of respiration, the RV systolic pressure increases, wheras the LV pressure decreases (called discordant ventricular interdependence), this finding is most consistent with constrictive physiology (Figure 1B). Ventricular interdependence has a sensitivity and specificity that far exceeds any other hemodynamic findings such as the square root sign and RV end diastolic pressure/RV systolic pressure ratio.^2^^,^^3^

## Pearls in Hemodynamics from editors Larry S. Dean, MD, and Morton J. Kern, MD

• Appropriate catheters should be used, which are properly placed and zeroed with simultaneous RV and LV pressure measurements during inspiration and expiration.
• In patients with atrial fibrillation, proper assessment should be paced.
• Elevated RV and LV pressures that vary together with respiration (ie, concordance) should be strongly considered as RCM.
• Elevated RV and LV pressures that are out of synchrony on inspiration (ie, discordant), wherein the RV pressure increases but the LV pressure decreases would support the diagnosis of CPD.

## Peer review statement

Section Editors Larry S. Dean and Morton J. Kern had no involvement in the peer review of this article and have no access to information regarding its peer review. Full responsibility for the editorial process for this article was delegated to Associate Editor Andrew M. Goldsweig.
